# 75. Fecal microbiome composition of cancer patients with infectious diarrhea varies by enteropathogen

**DOI:** 10.1093/ofid/ofaf695.026

**Published:** 2026-01-11

**Authors:** Adilene Olvera, Cynthia L Chappell, Kristi Hoffman, Kai Jiang, Christine Peterson, Lily Carlin, Matthew C Ross, Richard Gibbs, Donna Muzny, Sara Javornik Cregeen, Harshavardhan Doddapaneni, Joseph Petrosino, Pablo C Okhuysen

**Affiliations:** The University of Texas MD Anderson Cancer Center, Houston, Texas, Houston, TX; The University of Texas Houston School of Public Health, Houston, Texas; Baylor College of Medicine, Houston, Texas; The University of Texas MD Anderson Cancer Center, Houston, Texas; The University of Texas MD Anderson Cancer Center, Houston, Texas, Houston, TX; The University of Texas MD Anderson Cancer Center, Houston, Texas; Baylor College of Medicine, Houston, Texas; Baylor College of Medicine, Houston, Texas; Baylor College of Medicine, Human Genome Sequencing Center, HOUSTON, Texas; Baylor College of Medicine, Houston, Texas; Baylor College of Medicine, Houston, Texas; Baylor College of Medicine, Houston, Texas; The University of Texas MD Anderson Cancer Center, Houston, Texas

## Abstract

**Background:**

The importance of the gut microbiome in patients with cancer is increasingly recognized. We hypothesized that in cancer patients with infectious diarrhea, fecal microbiome composition is influenced not only by antibiotic or chemotherapy exposure, but also by underlying cancer diagnosis and the specific enteropathogen identified.

**Figure 1.**
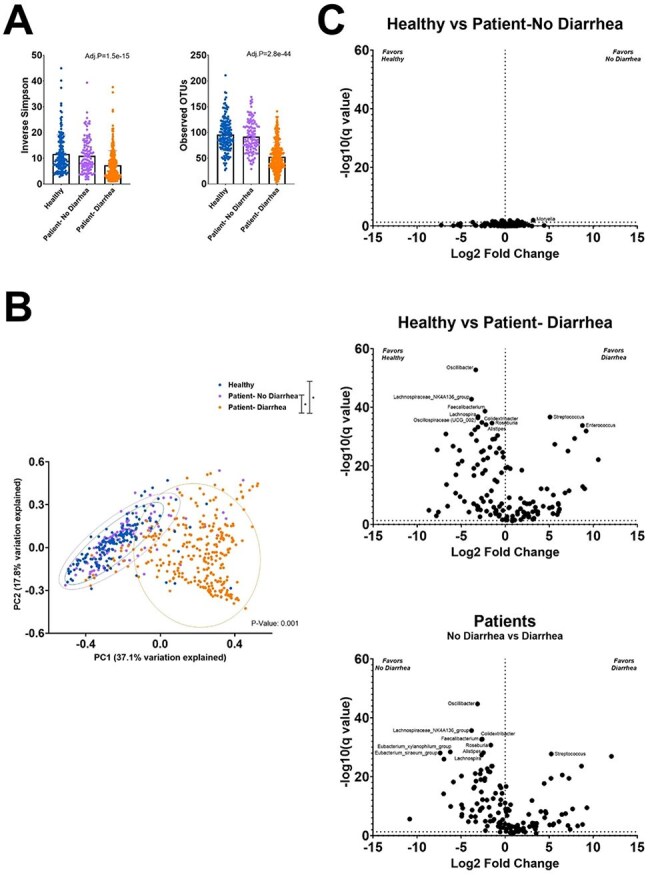
Fecal microbiome diversity and richness in healthy adults, patients with cancer and no diarrhea, and patients with cancer and infectious diarrhea Panel A. Alpha Diversity. Patients with cancer and infectious diarrhea in orange. Patient with cancer without diarrhea in purple and healthy adults in blue. Panel B. Beta diversity. Panel C Volcano plot with predominant taxa when comparing the three groups of patients.

**Figure 2.**
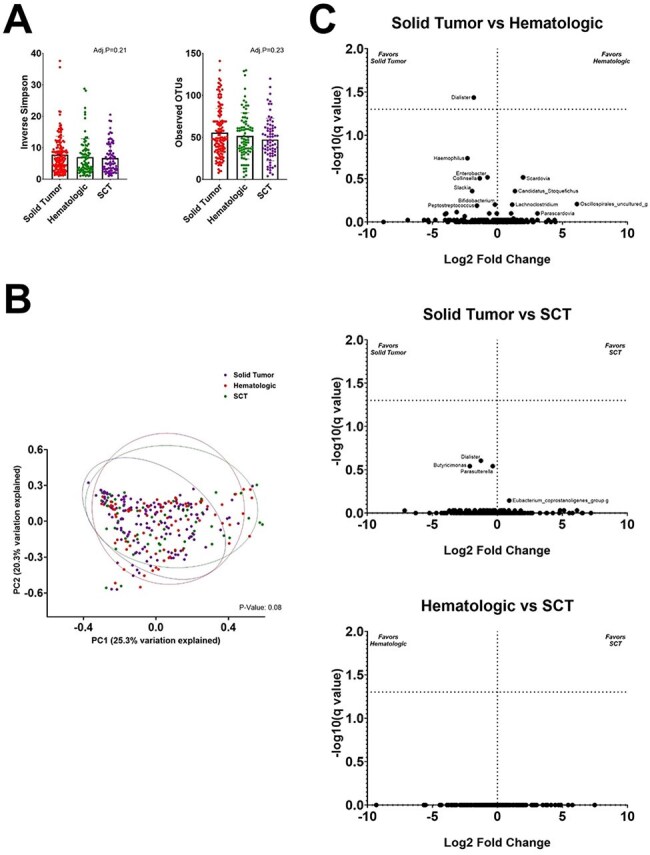
Fecal microbiome diversity and richness in patients with cancer and diarrhea according to underlying malignancy Panel A. Alpha diversity of fecal microbiome of patients with solid tumors in red, hematoloic malignancies (leukemia, lymphoma) in green and stem cell transplant in purple. Panel B. Beta diversity and Panel C. Volcano plot with predominant taxa

**Methods:**

We characterized the fecal microbiome (16Sv4 rRNA gene sequencing) of 298 cancer patients with diarrhea and an enteropathogen identified (BioFire, FilmArray). Controls included 113 cancer patients without diarrhea, and 167 healthy individuals. Microbiome diversity and taxonomic differences were assessed by Kruskal-Wallis and PERMANOVA. Results were adjusted for multiple comparisons.

**Figure 3.**
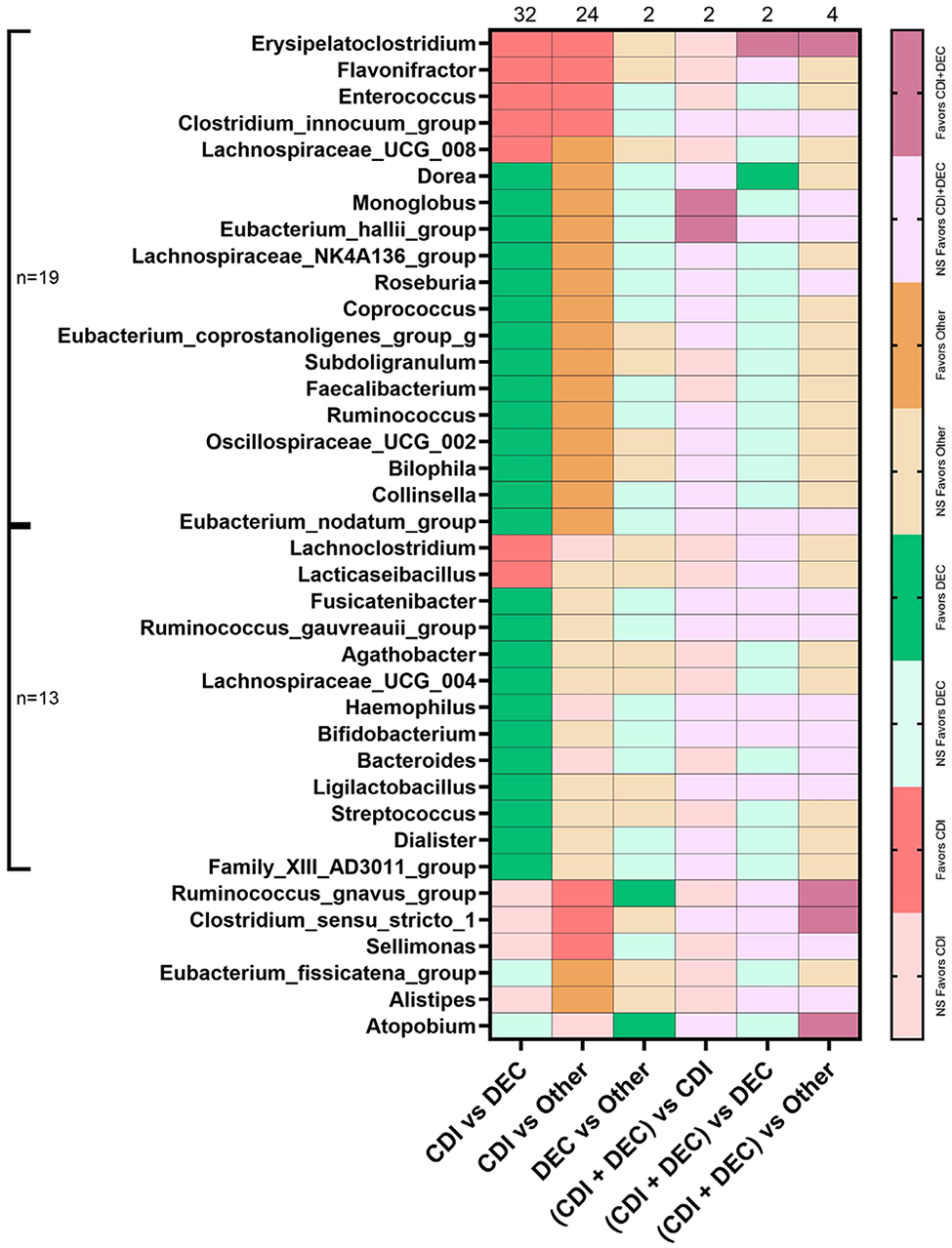
Summary table with comparisons of predominant taxa in patients with diarrhea due to CDI, DEC, and other pathogens. Summary table comparing predominant taxa according to pathogen identified. First column from the left shows 32 taxa with significantly differences when comparing CDI vs DEC, of these 7 were related to CDI and 25 with DEC. Second column from the left shows 24 taxa with significant differences when comparing CDI vs Other pathogens, of these 7 were related to CDI and 17 with infection due to other pathogens. Pink favors CDI, green favors DEC, orange favors other. NS denotes not significantly different.

**Results:**

The fecal microbiome differed significantly between cancer patients with diarrhea and both control groups. Diarrheal stools were less diverse and showed higher relative abundance of *Blautia, Enterococcus, Escherichia-Shigella,* and *Streptococcus.* In contrast, *Alistipes, Bacteroides, Faecalibacterium,* and *Oscillibacter* were more abundant in controls. Patients with solid tumors were less likely to receive antibiotics or immunosuppressants than patients with hematologic cancers, yet among those with diarrhea, microbiome composition did not differ significantly by cancer type, chemotherapy, immunosuppression, or antibiotic use ≤ 90 days. Enteropathogens identified included *Clostridioides difficile* (CDI, 53%), diarrheagenic *E. coli* (DEC, 16%), CDI coinfection (16%), and other pathogens (15%). When stratified by enteropathogen, specific shifts were evident: *Clostridioides* was enriched in patients with CDI, and *Escherichia-Shigella* in DEC. Excluding these pathogens, pairwise analysis further identified seven taxa significantly associated with CDI and 25 with DEC. Similarly, when comparing CDI to diarrhea due to other pathogens, 7 taxa were more abundant in CDI, while 17 were enriched in other infections—14 of which overlapped with DEC—suggesting taxa loss in CDI rather than uniquely gained elsewhere.

**Conclusion:**

In cancer patients with infectious diarrhea, the gut microbiome is disrupted in a pathogen-specific manner, largely independent of cancer type or recent exposure to antibiotics or immunosuppressants.

**Disclosures:**

All Authors: No reported disclosures

